# Assessment of Health-Related Quality of Life and Distress in an Asian Community-Based Cancer Rehabilitation Program

**DOI:** 10.3390/curroncol29100551

**Published:** 2022-09-27

**Authors:** Matthew Rong Jie Tay, Chin Jung Wong, Hui Zhen Aw

**Affiliations:** 1 Department of Rehabilitation Medicine, Tan Tock Seng Hospital, 11 Jalan Tan Tock Seng, Singapore 308433, Singapore; 2 Singapore Cancer Society Rehabilitation Center, 52 Jurong Gateway Rd, Singapore 608550, Singapore

**Keywords:** health-related quality of life, quality of life, patient-centered outcomes, patient-reported outcomes, distress, oncology, survivorship, rehabilitation, FACT-G7

## Abstract

Cancer survivors have reduced health-related quality of life (HRQOL) and high levels of distress during and after active treatment, due to physical, psychological, and social problems. Understanding the prevalence and associations of HRQOL and distress in a patient population in the community is important when designing rehabilitation programs. This was a cross-sectional observational study conducted at a community-based cancer rehabilitation center, with the aim of investigating the prevalence and associations of HRQOL and distress in cancer patients. There were 304 patients who were recruited. We found low levels of HRQOL and high levels of distress in patients, with a mean FACT-G7 total score of 11.68, and a mean distress thermometer score of 3.51. In the multivariate regression model, significant factors for low HRQOL were metastatic disease (*p* = 0.025) and Malay ethnicity (*p* < 0.001). Regression analyses also found that significant distress was associated with family health issues (*p* = 0.003), depression (*p* = 0.001), worry (*p* = 0.005), breathing (*p* = 0.007), getting around (*p* = 0.012) and indigestion (*p* = 0.039). A high prevalence of impaired HRQOL and distress was reported in cancer survivors even in a community rehabilitation setting. The physical and psychosocial well-being of cancer survivors should be monitored and managed as part of community-based cancer rehabilitation.

## 1. Introduction

Improving cancer mortality, quality of life and psychological well-being is increasingly important to cancer patients after treatment. A substantial number of cancer survivors have to deal with physical, psychological, and social problems during and immediately after active treatment, which reduces health-related quality of life (HRQOL) [1,2,3]. Although many of these problems may decline significantly with time, many patients still report impaired HRQOL scores which persist even after acute treatment has ceased [4]. As part of cancer survivorship, many of these patients will benefit from cancer rehabilitation services in the community to support the transition from hospital care to community living, and to return them to previous community activity levels [5,6]. Consequently, understanding the needs of these patients is important for the coordination and quality of outpatient care for this group of patients [7]. However, data on the HRQOL of cancer survivors undergoing community-based rehabilitation are currently lacking.

The overall burden of cancer diagnosis and treatment results in distress, which has been defined as a multifactorial, unpleasant emotional experience of a psychosocial, spiritual, and/or physical nature that may interfere with the ability to cope effectively with cancer, its physical symptoms, and its treatment [8]. Various studies have estimated that 10–40% of cancer survivors experience significant levels of distress [9,10,11]. Distress has a negative effect on patients and is associated with important aspects of cancer outcome including quality of life, performance status, treatment adherence, and healthcare utilization [12,13].

This has led to the adoption of the Distress Thermometer (DT), which is a brief visual analog scale for the routine assessment of distress and problems in cancer patients and has since been widely adopted and validated in multiple patient populations [14,15,16]. Additionally, the DT also incorporates a Problem List (PL) that lists specific causes of patient distress. Research on cancer survivorship is still in its infancy in Asia with reportedly high levels of distress. For example, a large Chinese cohort of cancer patients reported a prevalence rate of 20% using the DT [17]. In Korea, nearly a third of cancer patients were found to suffer from distress [18], with substantial proportions of cancer survivors in Southeast Asian countries also reporting psychological distress [19,20]. Consequently, this had led to the increasing awareness of cancer rehabilitation, which aims to address causes of psychological distress such as physical disability, pain, emotional distress, fatigue, or social functioning.

Therefore, the aim of this study was to investigate the prevalence and associations of HRQOL and distress in cancer patients.

## 2. Materials and Methods

### 2.1. Participants

This was a retrospective cohort study of Asian cancer survivors who had presented at a national community-based cancer rehabilitation center between 2018 to 2020. An individual was defined as a cancer survivor from the time of diagnosis through the balance of life, including those living with cancer and those free from cancer [21]. All cancer survivors were referred by clinical specialists or primary care physicians from any local healthcare institutions after they had completed their acute oncological treatment, i.e., chemotherapy, radiotherapy, surgery, or any combination of the aforementioned treatment. The referral criteria were patients who have completed active treatment and follow-up at hospitals, were in medically stable condition, and experienced symptoms that require rehabilitative support. The national outpatient community cancer rehabilitation program provides comprehensive rehabilitation services with a physician-led multidisciplinary team, which includes physiotherapists, occupational therapists, nutritionists, and medical social workers.

Eligible patients were adults ≥ 21 years old and enrolled in the rehabilitation program. Patients were excluded if they had terminally ill conditions or had major psychiatric illnesses. The clinical study was performed in accordance with the principles of the Declaration of Helsinki. Ethical approval was obtained from the local institutional review board, Agency for Integrated Care (2021-001).

### 2.2. Patient Evaluation

The primary outcome measure was HRQOL, as measured by the Functional Assessment of Cancer Therapy-General (FACT-G7) [22]—a short version of the general (FACT-G) questionnaire [23]. This is a rapid index of 7 high-priority FACT-G items which is used to evaluate symptom/concern burden and HRQOL in cancer patients over time, of which 3 items are from the physical well-being subscale of the FACT-G (fatigue, pain, and nausea), one item is from the emotional well-being subscale of the FACT-G (worry about condition worsening), and three items are from the functional well-being subscale (enjoyment of life, contentment with quality of life, and sleep). The FACT-G7 has demonstrated good validity and reliability in cancer samples [22,24]. The total score ranges from 0–28, with a score of 16 or lower indicating low HRQOL [25].

The DT and PL were completed by all cancer survivors during their initial screening visit. A DT is a single-item self-reported measure of distress. Patients were asked to grade their distress in the past week on an 11-point visual analog scale ranging from 0 (no distress) to 10 (extreme distress) [8]. A DT score of ≥5 was used as a cutoff indicating a clinically significant level of distress [26,27].

The DT is accompanied by the PL, which consists of 34 problems commonly experienced by cancer patients. These problems are grouped into 5 categories: spiritual/religious concerns, practical problems, family problems, emotional problems, and physical problems. Respondents are instructed to indicate (yes or no) if any of the items listed have been a problem in the past week [8].

Additional socio-demographic and clinical data were collected from patient records.

### 2.3. Statistical Analysis

Distribution of sociodemographic and clinical data was presented with appropriate descriptive statistics. The distributions of categorical and continuous variables were compared using chi-squared test and independent t-test, respectively.

To analyze the association between socio-clinical factors and HRQOL, we used a multivariable logistic regression model. These models adjusted for the covariates of age, gender, ethnicity, tumor type, and cancer stage.

We also constructed a logistical regression model of all thermometer items to determine the contribution of each item to the presence of clinically significant distress (defined as DT of ≥5), after adjusting for significant socio-clinical variables.

All estimates were reported along with the 95% confidence interval (CI). Statistical analyses were performed using SPSS version 26.0 (IBM Corp., Armonk, NY, USA). All statistical tests were performed with alpha set at 0.05.

## 3. Results

We screened 311 patients, of which 7 patients were excluded as they had terminally ill conditions. The baseline characteristics of the 304 study participants are shown in Table 1. The majority were female patients (80.6%) and of Chinese ethnicity (92.1%). The five most common cancer types were breast cancers (59.5%), prostate cancers (9.9%), colorectal cancers (6.3%), hematological malignancies (4.6%), and ovarian cancers (4.3%).

The mean FACT-G7 total score was 11.68 (SD = 4.17), with 267 participants (87.8%) displaying a low FACT-G7 score. The mean distress thermometer score was 3.51 (SD = 2.45) with 188 (61.8%) of participants indicating significant distress (rated as ≥5) (Table 2).

On univariate analysis, ethnicity, and cancer stage were significantly associated with HRQOL. In the multivariate analysis, those of a Malay ethnicity were less likely to have a low HRQOL (OR = 0.157; 95% CI = 0.056–9.44; *p* < 0.001). Conversely, those with metastatic disease were more likely to have a low HRQOL (OR = 2.98; 95% CI = 1.15–7.72; *p* = 0.025) (Table 3).

The mean number of problems on the PL was 7.21 (SD = 5.06), with common causes being worry (48.0%), fatigue (47.4%), memory/concentration (46.7%), tingling in hands/feet (46.4%) and sleep (42.8%) (Table 4).

Preliminary analyses were performed to identify potential significant clinical variables (age, gender, ethnicity, tumor type, and cancer stage) influencing the presence of significant distress. Risk factors for significant distress were a female gender (OR 2.57; CI = 1.32–5.00; *p* = 0.005) and hematological malignancies (OR 0.296; 0.108–0.808; *p* = 0.018).

A logistic regression model was created to determine problem list items associated with significant distress, adjusted for gender and hematological malignancies. We found that participants with significant distress were more likely to indicate problems with family health issues (OR = 4.27; 95% CI = 1.41–10.71; *p* = 0.003), depression (OR = 8.38; 95% CI = 2.51–28.04; *p* = 0.001), worry (OR = 3.08; 95% CI = 1.41–6.75; *p* = 0.005), breathing (OR = 4.59; 95%CI = 1.52–13.86; *p* = 0.007), getting around (OR = 4.03; 95% CI = 1.36–11.95; *p* = 0.012) and indigestion (OR = 3.31; 95%CI = 1.06–10.32; *p* = 0.039) (Table 5).

## 4. Discussion

We found a low mean FACT G-7 HRQOL score in our study population, with the large majority of patients reporting a low score of ≤16. Our baseline evaluation indicates that our cancer survivors who are accessing community-based rehabilitation still have persistently low HRQOL, which likely reflects persistent impairments even after the acute cancer treatment phase. Our results also confirm that lack of energy, pain, and sleep issues are persistent and bothersome symptoms in cancer survivorship [28]. The majority of the FACT-G7 items are also derived from the FACT-G physical and functional well-being scales, which highlight areas of impairment in these domains. Similarly, a study on Singaporean breast cancer survivors also reported low composite HRQOL scores with high levels on multiple symptom domains, even at in patients up to 5 years after breast cancer [29].

We also found tumor stage and ethnicity to have significant associations with HRQOL. Malay patients may be likely to have a better HRQOL than Chinese patients [30]. This may be due to Chinese patients having more unmet supportive care needs based on a study at an outpatient oncology clinic in a tertiary hospital in Malaysia [31]. Additionally, a prospective longitudinal observational study completed at a regional hospital in Singapore found that ethnicity was a significant determinant of HQOL in the domains of role limitations due to emotional problems and social functioning on the Short Form-36 health survey scale [32]. It is also possible that this may be due to Malays displaying a higher mental health component than the Chinese, which has been shown in a local multi-ethnic study on chronic illnesses [33]. This may be related to differences among ethnic groups in terms of culture, identity, or minority status [34]. However, this finding requires cautious interpretation given the small number of Malay patients in this study. Further studies are also required to understand the relationship between ethnicity and HRQOL in cancer patients, and if this influences patients’ response to disease and treatment sequelae.

In a study performed in Shanghai, China, and Houston, US, a comparison of HQOL was performed between Chinese and American breast cancer survivors. Chinese patients were found to have a lower average HQOL score, along with lower levels of functional, physical, social, and emotional well-being compared to American women [35]. Possible causes include less aggressive symptom control strategies, lower income, and greater use of chemotherapy which may account for country differences in QOL. Another study by Lam et al. also found that Chinese women with breast cancer had more unmet needs compared with German women, with more unmet needs in the health system and information domains [36]. Our findings of a low HQOL score in Asian patients suggest that clinicians should pay more attention to the unmet needs and address social support barriers in cancer survivors. However, further studies are indicated to determine if culture, education, religion, access to rehabilitative services and socioeconomic support account for differences between Asian and Western cancer survivors.

The presence of metastatic disease was associated with poorer HRQOL. These patients usually require a more complicated course of treatment, resulting in greater physical and psychological sequelae [37,38,39]. Patients with bone metastases have a higher incidence of pain, while those with visceral metastases require multiple lines of treatment [40]. The presence of metastases is also associated with depression and overall HRQOL, which may be due to the fear of additional disease spread and the burden of additional treatments [41]. Despite the presence of metastases, select patients can still benefit from careful mobility and strength-based interventions, to maintain existing function and HRQOL [42].

We also report a mean distress level of 3.51, which is similar to an earlier study in Singapore, which reported a mean distress level of 3.3 [20]. Nearly 2/3 of our study population experiencing significant distress, which is in line with a reported worldwide prevalence rate of 20–52% of cancer patients [43,44]. Several physical symptoms including breathing, getting around and indigestion were associated with significant distress. These may be reflective of limited mobility and activities and daily living, leading to unmet supportive care needs and high dependence on others [44]. The results of our study also indicated that psychosocial and physical problems were important factors in determining if a patient experienced significant distress. In many of these patients, depression, worry, and family health issues were significantly associated with distress, with nearly half of the study population expressing worry. Unsurprisingly, distress has been shown to be strongly associated with mood disturbances including stress, anxiety, and depression which may not reach the criteria for clinical diagnosis, but still interfere with HRQOL [45,46]. Our findings emphasize the need to consistently screen for social and emotional issues in cancer survivors, even while many of these patients are undergoing physical therapy-based rehabilitation [20].

Cancer rehabilitation has been described as an important component of cancer survivorship, through addressing the side effects of cancer and cancer treatment. Decreased cardiorespiratory fitness is a common feature in cancer patients, which can be contributed by bed rest and deconditioning, radiation therapy involving the chest wall, or the pharmacological management of cancers [5]. Physical therapy-based interventions have been shown to reduce cancer-related fatigue, and improve HROL and aerobic fitness, which in turn leads to increased ADL performance [47,48]. Multidisciplinary and individualized therapy involving physical training, patient education, and psychological interventions have been also shown to reduce anxiety and distress and improve HRQOL [49,50]. Our findings from this study highlight the unmet physical and psychosocial needs of cancer survivors and reinforce the need for multidisciplinary post-acute cancer rehabilitation pathways as part of quality cancer care. However, further studies are required to determine predictors of rehabilitative success and methods for integrating rehabilitative pathways as part of the cancer survivorship continuum.

This study has several limitations. Firstly, this was a cross-sectional study, which limits the evaluation of a causal relationship between clinical factors and HRQOL or distress. Second, these findings may only be generalizable to patients presenting at a community rehabilitation center, and hence physical disability and HRQOL may be more severe in patients who do not have access to such services. Third, we chose the FACT G7 due to its brevity and ease of administration. We did not use other more complex scales such as the FACT-G or the European Organization for Research and Treatment of Cancer Core Quality of Life Questionnaire (EORTC QLQ-C30). The FACT G7 may not reflect social and emotional aspects of HRQOL as much as symptom-related concerns [51], although this is attenuated to a certain extent by the use of the DT in our study. Fourth, we also did not collect information such as comorbidities, socio-economic status, social support, or self-efficacy in symptom management, which have previously been reported to affect overall well-being [52]. Fifth, a limitation of this study was that the increase in familywise error rate across the reported statistical analyses was not controlled. Therefore, we consider our findings relatively preliminary. Lastly, we recruited patients with various cancer types and stages to reflect the real-life challenges of cancer survivors in the general population. However, the needs of cancer survivors may be unique in certain cancer types/stages, and individualized and tailored rehabilitative interventions are often required.

## 5. Conclusions

We report a significant proportion of patients with impaired HRQOL presenting at a first visit at an Asian community rehabilitation center, with practical and emotional problems playing a key role. This underscores the need for early identification of the physical and psychosocial needs of cancer survivors, so that a holistic and multi-focused rehabilitation program can enhance their HRQOL.

## Figures and Tables

**Table 1:** Demographic and clinical characteristics of the study participants (*N* = 304).

Characteristics	
Age, mean (SD)	
- 31–40	12 (3.9)
- 41–50	23 (7.6)
- 51–60	88 (28.9)
- 61–70	112 (36.8)
- 71–80	69 (22.7)
Gender, *n* (%)	
- Male	59 (19.4)
- Female	245 (80.6)
Ethnicity, *n* (%)	
- Chinese	280 (92.1)
- Malay	18 (5.9)
- Indian	6 (2.0)
Cancer type, *n* (%)	
- Breast	181 (59.5)
- Brain	4 (1.3)
- Bladder	4 (1.3)
- Colorectal	19 (6.3)
- Head and neck	9 (3.0)
- Hepatopancreatobiliary	3 (1.0)
- Kidney	4 (1.3)
- Lung	12 (3.9)
- Hematological	14 (4.6)
- Ovarian	13 (4.3)
- Prostate	30 (9.9)
- Uterine	11 (3.6)
Stage, *n* (%)	
- No recurrence	144 (47.3)
- Localized/ regional	52 (17.1)
- Metastatic	108 (35.5)

**Table 2:** FACT-G7 and distress scores (*N* = 304).

Scores	
FACT-G7 total scores, mean (SD)	11.68 (4.17)
FACT-G7, *n* (%)	
- Low (≤16)	267 (87.8)
- Not low (>17)	37 (12.2)
FACT-G7 mean item ratings, mean (SD)	
- I have a lack of energy (fatigue)	1.49 (1.11)
- I have nausea	1.43 (1.24)
- I have pain	0.34 (1.28)
- I worry that my condition will get worse	1.56 (1.28)
- I am able to enjoy life	2.26 (1.30)
- I am sleeping well	2.31 (1.15)
- I am content with the quality of my life right now	2.30 (1.15)
Distress thermometer, mean (SD)	3.51 (2.45)
Distress thermometer ≥ 5, *n* (%)	188 (61.8)

**Table 3:** Associations with low health-related quality of life scores (*N* = 304).

	Univariate Analysis	Multivariate Analysis		
Variables	*p* Value	Odds Ratio	95% CI	*p* Value
Age	0.441			
Gender				
- Male	Reference			
- Female	0.601			
Ethnicity				
- Chinese	Reference	Reference	-	-
- Malay	<0.001	0.157	0.056–9.439	<0.001
- Indian	0.598	0.488	0.052–4.55	0.529
Tumor type	0.873			
Cancer stage				
- No recurrence	Reference	Reference	-	-
- Localized/ regional	0.921	1.11	0.446–2.78	0.818
- Metastatic	0.014	2.98	1.15–7.72	0.025

**Table 4:** Top 10 issues in problem list (*N* = 304).

Problems, *n* (%)	
Worry	146 (48.0)
Fatigue	144 (47.4)
Memory/concentration	142 (46.7)
Tingling in hands/feet	141 (46.4)
Sleep	130 (42.8)
Pain	129 (42.4)
Skin dry/itchy	117 (38.4)
Fears	91 (29.9)
Insurance	69 (22.7)
Sadness	68 (22.4)

**Table 5:** Significant problems associated with distress (*N* = 304).

Variables	Odds Ratio	95% CI	*p* Value
Practical problems			
Family health issues	4.27	1.41–10.71	0.003
Emotional problems			
Depression	8.38	2.51–28.04	0.001
Worry	3.08	1.41–6.75	0.005
Physical problems			
Breathing	4.59	1.52–13.86	0.007
Getting around	4.03	1.36–11.95	0.012
Indigestion	3.31	1.06–10.32	0.039

## Data Availability

The data presented in this study are available on request from the corresponding author.
